# Validation of the Medical Research Council prion disease rating scale in France

**DOI:** 10.1093/braincomms/fcad267

**Published:** 2023-11-08

**Authors:** Jean-Philippe Brandel, Arlette Welaratne, Angeline Denouel, Stéphane Haïk

**Affiliations:** AP-HP, Cellule Nationale de Référence des MCJ, Salpêtrière Hospital, Paris F-75013, France; Institut du Cerveau et de la Moelle épinière, ICM, Paris F-75013, France; Inserm, Paris F-75013, France; AP-HP, Cellule Nationale de Référence des MCJ, Salpêtrière Hospital, Paris F-75013, France; Institut du Cerveau et de la Moelle épinière, ICM, Paris F-75013, France; Inserm, Paris F-75013, France; AP-HP, Cellule Nationale de Référence des MCJ, Salpêtrière Hospital, Paris F-75013, France; Institut du Cerveau et de la Moelle épinière, ICM, Paris F-75013, France; Inserm, Paris F-75013, France

**Keywords:** prion disease, MRC scale, evaluation, therapeutic studies

## Abstract

The development of assessment tools other than survival time is necessary to conduct therapeutic trials in prion diseases (also known as subacute transmissible encephalopathies). The Medical Research Council Prion Disease Rating Scale published by Thompson *et al*. (The Medical Research Council prion disease rating scale: A new outcome measure for prion disease therapeutic trials developed and validated using systematic observational studies. *Brain*. 2013; 136: 1116–27.) is the first attempt at a specific evaluation of prion diseases to avoid the floor effect seen in other scales. Validation of this scale in other countries is essential because, given the rarity of these diseases, therapeutic trials are likely to be multi-centre and international. After translation into French, we assessed by phone 173 cases classified as sporadic Creutzfeldt–Jakob disease out of 852 patients notified to the French national surveillance network between November 2014 and May 2021. Data showed that the natural history of the disease is similar in the UK and France. Patients who have a heterozygous genotype at codon 129 of the prion protein gene have a slower decline than homozygous patients. In rapidly progressing patients, death occurs shortly after reaching a low score or after a ‘pre-terminal plateau’ at a very low score. The similarities of disease progression profile observed in France and the UK with somewhat different surveillance systems and by distinct procedures highlight the robustness of the Medical Research Council Prion Disease Rating Scale that can be thus used to define primary endpoints of future trials at the international level.

## Teaser

Brandel *et al*. administered a translated version of the Medical Research Council Prion Disease Scale by telephone and observed a similar decline in patients with sporadic Creutzfeldt–Jakob disease in France and the UK. This result validating the scale opens the possibility of using it as an evaluation criterion for international therapeutic trials.

## Graphical Abstract

**Graphical abstract:**
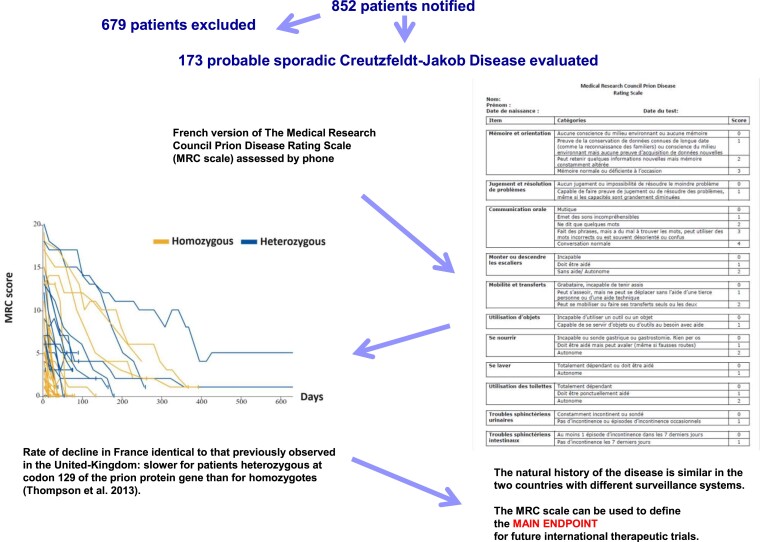

## Introduction

Prion diseases (also known as transmissible subacute encephalopathies) are fatal neurodegenerative diseases caused by the misfolding of the host-encoded prion protein. The most common form of transmissible subacute encephalopathies in humans is sporadic Creutzfeldt–Jakob disease (sCJD). There are also genetic transmissible subacute encephalopathies and acquired forms of CJD such as iatrogenic or variant CJD. No effective treatment is currently available. Therapeutic trials are feasible but challenging given the rarity of these diseases, which explains the lack of interest of the pharmaceutical industry in the development of prion-specific drugs. This leads to testing molecules already available on the market such as quinacrine and doxycycline. On the other hand, the rapid evolution of most forms of CJD explains the difficulty for families to consent to the inclusion of patients in placebo controlled clinical trials. Another difficulty is related to the lack of efficient evaluation tools for measuring the positive effect of tested drugs on disease course and patient quality of life. To date, with the exception of one,^1^ survival time has been used as the main endpoint in all clinical trials in the field.^2,3^ Death is a clearly defined and unambiguous event, but its use as a primary endpoint is problematic since survival can be strongly influenced by end-of-life care (enteral feeding, intensive care, etc.) that may vary significantly between countries. Survival gives no indication in disease course and patient’s functional status. The functional scales usually employed to monitor patients with CNS disorders are of little relevance in CJD because of likely floor effect. This is the reason why Thompson *et al*.^4^ have provided a specific scale (Medical Research Council Prion Disease Rating Scale or MRC scale) assessing five domains including cognitive function, speech, mobility, personal care and feeding and continence to evaluate the rate of decline observed in all forms of prion diseases. They proposed to use it as a central tool to define primary endpoints in future clinical trials in prion diseases.^4^ Validation of the MRC scale in other countries with developed surveillance systems but distinct CJD detection and care modalities is therefore pivotal to set up future international clinical trials.

## Material and methods

**Figure 1:**
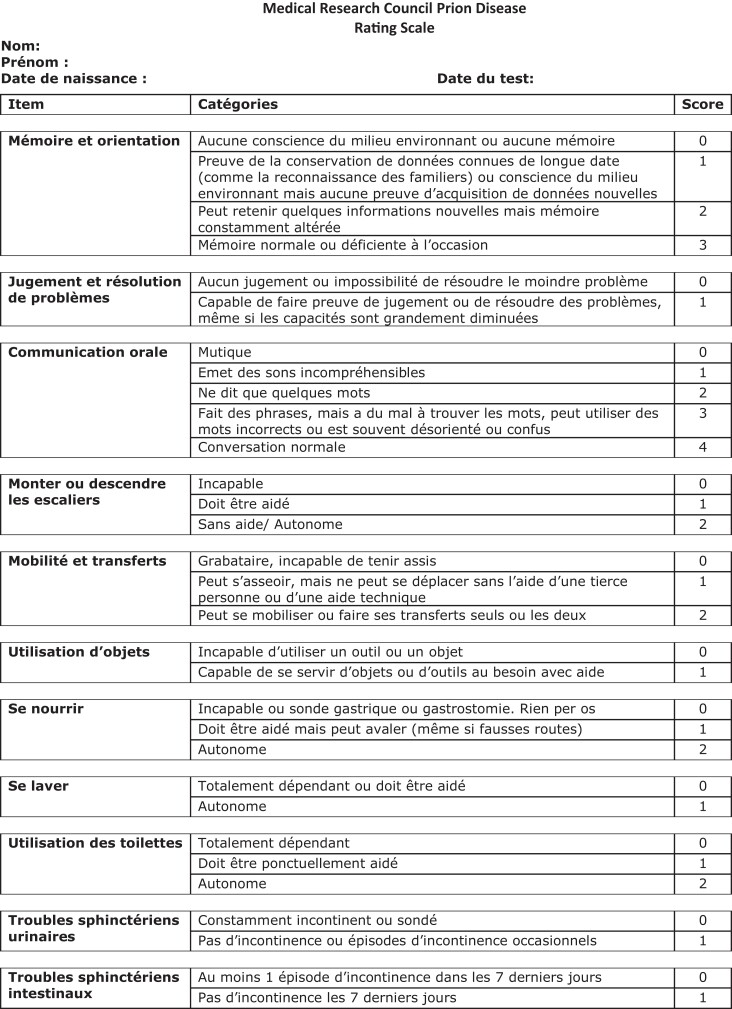
MRC scale: French version.

We first translated the MRC scale into French (Fig. 1). To make the scale easier to use, the order of the questions has been changed from the scale proposed by Thompson *et al*.^4^ Our questionnaire begins with the evaluation of cognitive functions and language, then mobility, followed by feeding and personal care and finally continence. The item regarding the use of tools was placed after those on cognition and mobility because this item has both cognitive and motor aspects.

Between November 2014 and May 2021, all patients reported to the national CJD surveillance network could potentially be included in the study. Some patients among those meeting diagnostic criteria for probable CJD or with progressive neurological syndrome and at least one EEG showing periodic sharp wave complexes or positive detection of 14-3-3 protein in the CSF or typical high signals on brain MRI at the time of notification were evaluated and followed up. All these patients were contacted by the same clinical research associate (A.W.). Given the organization of the French surveillance network for CJD, which collects clinical files of patients from neurology teams directly in charge of the patients and whose neurologists only travel to see patients suspected of or suffering from variant CJD, the assessment was performed only by phone at inclusion and then every 2 weeks until the score reached 0 on the MRC scale.

## Ethics statement

This observational study is non-interventional and is based on data from the national CJD surveillance network, which has received permanent authorization from the Commission Nationale Informatique et Libertés: CNIL authorization n° 900038 of the 20 June 2000.

## Results

From November 2014 to May 2021, a total of 852 patients consecutively notified to the French surveillance network were initially assessed. For 605 patients, no follow-up was possible after the first phone contact for various reasons: patient already deceased (*N* = 120) or with a too advanced disease (*N* = 4), patient having another diagnosis (*N* = 107), difficulty to contact a person from the nursing staff (*N* = 242) and discharged from hospital (*N* = 132). Among the 124 patients who have already died or with a too advanced disease on first contact, there were three genetic CJD (two with E200K mutation and one with V210I mutation) and 82 sporadic CJD (26 certain, 54 probable, and 2 possible) with a significantly shorter duration of evolution (mean: 3.6 months) than the 173 cases finally included in the study (mean: 6.6 months) and a higher proportion of homozygous patients. The remaining 39 patients were not classified as CJD. Finally, 247 patients were able to have at least one evaluation with the MRC scale. Among these patients, 173 were classified as sporadic CJD (49 definite, 122 probable, and 2 possible), six as genetic and one as probable CJD after growth hormone treatment. A total of 64 patients were classified with an alternative diagnosis, and three were not classified. Due to the small number of genetic or iatrogenic cases, validation of the scale only focused on sporadic forms of the disease. Data for sporadic CJD cases are summarized in Table 1. Patients with a diagnosis of sCJD had a mean number of assessments of 4 (1–49).

**Table 1:** Characteristics of sporadic CJD patients with at least one evaluation with the MRC scale

	Definite CJD (49)	Probable CJD (122)	Possible CJD (2)
Gender (% women)	69.39	55.74	100.00
Mean time between disease onset and first MRC score (days)	102 (18–798)	113 (8–765)	241 (78–404)
Mean duration of disease (months)	6.3 (1–47)	6.7 (2–33)	19.5 (15–24)
Codon 129 homozygous patients	21	48	2
Codon 129 heterozygous patients	9	16	0
Periodic sharp wave complexes at EEG	8	17	0
Positive detection of 14-3-3 in CSF	41	97	0
Typical high signals on brain MRI	39	97	0

**Figure 2:**
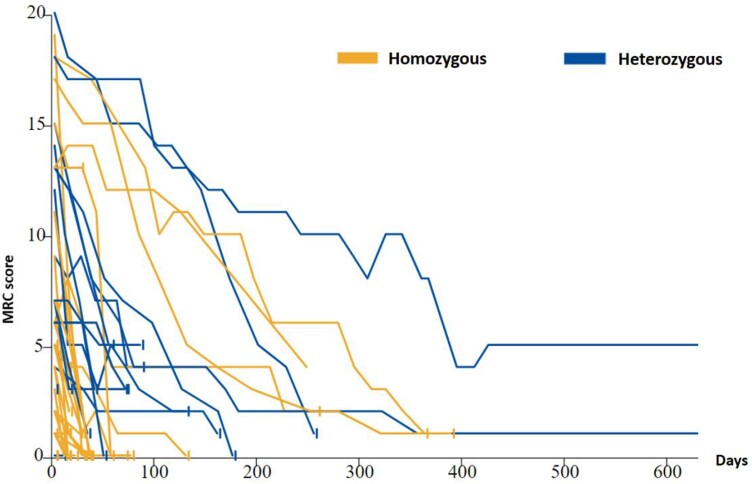
**Trajectories of cases with probable or definite CJD according to genotype at codon 129 of the *PRNP* gene up to 600 days of post-inclusion in France.** The cases are (homozygous methionine or valine; heterozygous methionine–valine).^4^

**Figure 3:**
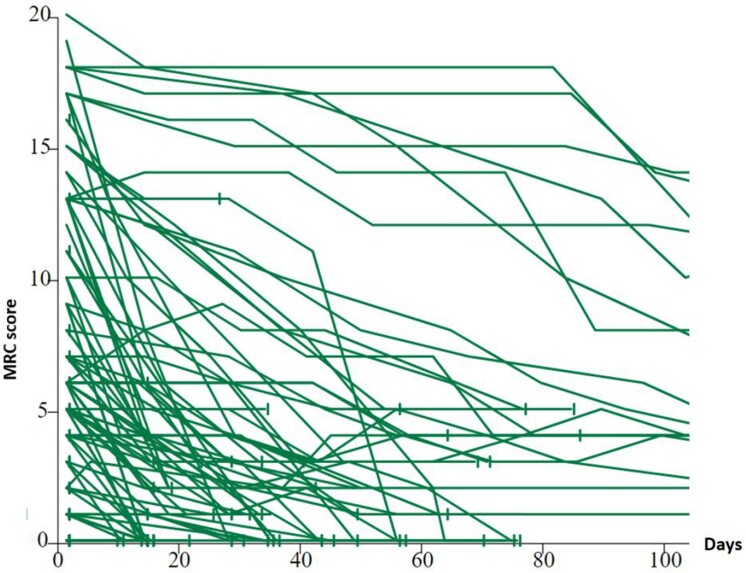
**Trajectories of cases with probable or definite sCJD for the first 100 days in France: curve similar to that observed in the UK.** ^4^

The mean time between disease onset and the first MRC score was just over 3 months for definite and probable sCJD, while the total duration of disease progression was 6.3 and 6.7 months. A total of 94 patients (54%) had a *PRNP* gene study with, among them, 69 (73%) homozygotes met/met or val/val at codon 129 and 25 (27%) heterozygotes met/val (Table 1). At the first contact, 10% of sCJD patients had a score on MRC scale between 20 and 15, 12% between 14 and 10 and 53% between 9 and 1%, and 25% had a score equal to 0. The majority of cases between 0 and 9 were met/met at codon 129, while cases between 15 and 10 were met/val or val/val. Natural disease progression was heterogeneous. Patients with CJD who are heterozygous at codon 129 had a slower decline than homozygotes (Fig. 2). In patients with a rapid progression profile, death occurs shortly after reaching a low score or after a ‘pre-terminal plateau’ at a very low score (Fig. 3).

## Discussion

Case monitoring was only possible in a minority of patients for different reasons. The most frequent reason was the difficulty of reaching a person from the nursing staff or because the patient had already been discharged from the hospital at the time of the first contact. This situation is reminiscent of the one we observed in a randomized trial that aimed at evaluating the effect of doxycycline versus placebo in which more than 70% of patients were excluded before inclusion.^1^ In addition, 18% of the patients had died at first contact, which is comparable with the 14% of patients who died before inclusion in the doxycycline trial.^3^ Of these patients, the majority (82/124) were particularly rapidly progressive cases of CJD. Among patients evaluable by the MRC scale, the majority are already in serious condition at the first assessment with a score on the MRC scale below 10 with even 25% of patients at the terminal stage of the disease and a score equal to 0. Only 22% of patients have a score greater than 10 reflecting a not too advanced clinical state. These results are consistent with what had been observed during the doxycycline trial in France where only a small proportion of the patients notified to the surveillance network could be included, with an over-representation of patients heterozygous at codon 129. This highlights the difficulty of making a diagnosis early enough to be able to include patients in a therapeutic protocol and assess them with a scale specifically designed to reduce the floor effect.

On the other hand, we confirm that the MRC scale is easily usable by phone, allowing it to be assessed as often as desired to monitor the rapid evolution typical of sporadic CJD.

As it was described in the UK, most French patients with sporadic CJD showed a rapid decline over weeks or a few months, and few patients a rapid decline followed by a ‘pre-terminal’ plateau at very low functional levels (Fig. 3). *PRNP* codon 129 homozygous (methionine–methionine or valine–valine) French CJD patients declined more rapidly than heterozygous ones as it was also observed in the UK cohort (Fig. 2). Among the patients for whom a study of the *PRNP* gene was possible, 27% were heterozygous, which represents a higher frequency than that usually observed in sCJD.^5^ This was due to the fact that the majority of very rapidly evolving cases were homozygous at codon 129 and could not be included.

The comparable disease progression profile obtained in two countries with somewhat different CJD surveillance systems shows that the MRC scale is a robust tool that could play a key role in defining the primary endpoint (such as the rate of decline) of future international therapeutic studies on prion diseases and notably in sporadic CJD. One of the advantages of using the evolution of MRC scale scores as the main criterion in future therapeutic studies is the reduced sensitivity to the nursing care provided to the patient at the end of life. This is different from death, which is problematic as an endpoint for clinical trial since survival can be strongly influenced by end-of-life care and provides limited information on the effect of treatment on the course of the disease and its impact on patient quality of life. In contrast, the use of a functional scale specifically designed for CJD patients monitoring takes into account the clinical state of the patient at inclusion and should be able to demonstrate the effectiveness of a treatment in slowing down CJD evolution or improving patient functional scores before fatal outcome.

Our data also suggest that the natural history of sporadic CJD is similar in France and the UK, two countries with a comparable level of care support but different systems of surveillance, clearly supporting the relevance of building future trials at the international level, a key issue in such a rare pathological condition.

## Data Availability

The data can be made available to interested research groups upon request.
